# Poly[hexa­aqua­bis­(μ_3_-hepta­nedioato-κ^3^ *O*:*O*′:*O*′′)dimagnesium]

**DOI:** 10.1107/S1600536811015492

**Published:** 2011-05-07

**Authors:** Xia-Xia Guo, Jian-li Lin

**Affiliations:** a Center of Applied Solid State Chemistry Research, Ningbo University, Ningbo, Zhejiang 315211, People’s Republic of China

## Abstract

In the title compound, [Mg_2_(C_7_H_10_O_4_)_2_(H_2_O)_6_]_*n*_, the Mg^II^ ion is coordinated by three aqua ligands and three O atoms from three heptanedioato ligands in a distorted octa­hedral geometry. Each heptanedioato ligand bridges three Mg atoms, generating polymeric layers parallel to the *bc* plane. The polymeric layers related by translation along the *a* axis inter­act further *via* O—H⋯O hydrogen bonds, which consolidate the crystal packing.

## Related literature

For general background to microporous coordination polymers, see: Borkowski & Cahill (2006 ▶); Dimos *et al.* (2002 ▶); Kim *et al.* (2001 ▶). For related structures, see: Liu *et al.* (2009 ▶).
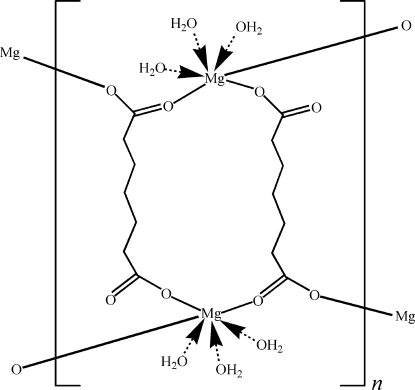


## Experimental

### 

#### Crystal data

[Mg_2_(C_7_H_10_O_4_)_2_(H_2_O)_6_]
*M* *_r_* = 236.51
Monoclinic, 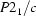

*a* = 14.311 (3) Å
*b* = 8.2080 (16) Å
*c* = 9.1280 (18) Å
β = 96.22 (3)°
*V* = 1065.9 (4) Å^3^
*Z* = 4
Mo *K*α radiation
μ = 0.18 mm^−1^
*T* = 293 K
0.1 × 0.1 × 0.1 mm

#### Data collection

Rigaku R-AXIS RAPID diffractometer
Absorption correction: multi-scan (*ABSCOR*; Higashi, 1995 ▶) *T*_min_ = 0.982, *T*_max_ = 0.982
8118 measured reflections
1880 independent reflections
1440 reflections with *I* > 2σ(*I*)
*R*_int_ = 0.040

#### Refinement

*R*[*F* ^2^ > 2σ(*F* ^2^)] = 0.046
*wR*(*F* ^2^) = 0.121
*S* = 1.16
1880 reflections
136 parameters
H-atom parameters constrained
Δρ_max_ = 0.34 e Å^−3^
Δρ_min_ = −0.46 e Å^−3^

### 

Data collection: *RAPID-AUTO* (Rigaku, 1998 ▶); cell refinement: *RAPID-AUTO*; data reduction: *CrystalStructure* (Rigaku/MSC, 2004 ▶); program(s) used to solve structure: *SHELXS97* (Sheldrick, 2008 ▶); program(s) used to refine structure: *SHELXL97* (Sheldrick, 2008 ▶); molecular graphics: *SHELXTL* (Sheldrick, 2008 ▶); software used to prepare material for publication: *SHELXL97*.

## Supplementary Material

Crystal structure: contains datablocks global, I. DOI: 10.1107/S1600536811015492/cv5076sup1.cif

Structure factors: contains datablocks I. DOI: 10.1107/S1600536811015492/cv5076Isup2.hkl

Additional supplementary materials:  crystallographic information; 3D view; checkCIF report

## Figures and Tables

**Table 1:** Hydrogen-bond geometry (Å, °)

*D*—H⋯*A*	*D*—H	H⋯*A*	*D*⋯*A*	*D*—H⋯*A*
O5—H5*C*⋯O2^i^	0.84	1.92	2.741 (3)	165
O5—H5*D*⋯O6^ii^	0.84	1.99	2.818 (3)	168
O6—H6*C*⋯O2^iii^	0.82	2.07	2.882 (3)	170
O6—H6*D*⋯O5^iii^	0.82	2.06	2.879 (3)	171
O7—H7*A*⋯O4^iv^	0.83	1.94	2.725 (3)	158
O7—H7*B*⋯O2^v^	0.79	2.26	2.798 (3)	127

Symmetry codes: (i) 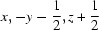; (ii) 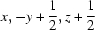; (iii) 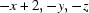; (iv) 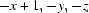; (v) 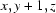.

**Fig. 1.**
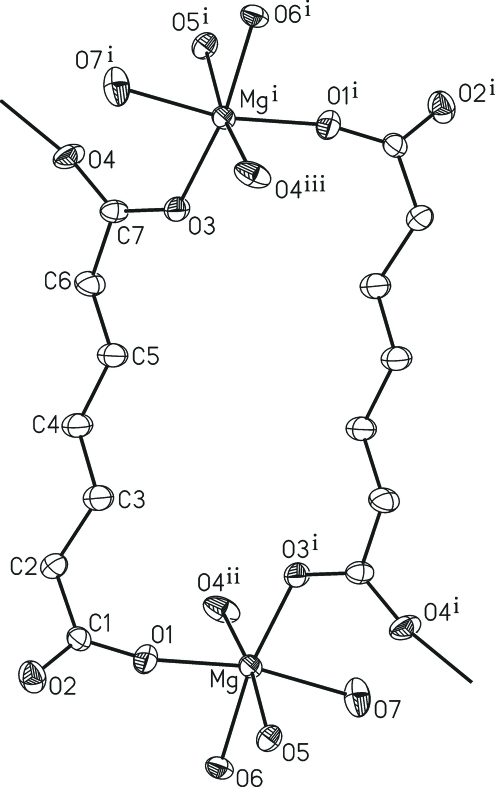
A portion of the crystal structure of (I) showing the atomic numbering and 45% probabilty dispalcement ellipsoids [wymmetry codes: (i)-x + 1, -y, -z;(ii)-x + 1, y + 1/2, -z - 1/2;(iii)-x + 1, y - 1/2, -z - 1/2.]

**Fig. 2.**
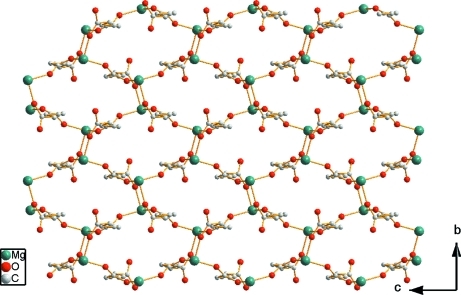
Two-dimensional polymeric layer in (I) viewed along the axis a.

## supplementary crystallographic information

### Comment

The past decade has witnessed enormous expansion of research on robust microporous coordination polymers (Borkowski *et al.*, 2006; Dimos *et al.*, 2002; Kim *et al.*, 2001). For such purpose, design and synthesis of novel coordination polymers have been focused on organic ligands, others have reported lists of complexs used dicarboxylic acids. In this contribution, we report the crystal structure of the title compound (I).

In (I) (Fig. 1), two carboxylate groups of pimelato (pim^2–^) ligand display different coordination behaviour - in the O1–C1–O2 group only one O1 atom coordinate one Mg center, while the O3–C7–O4 carboxylate group coordinate two Mg centers in an *syn*/anti mode. The Mg atoms are six-coordinated by three oxygen atoms from three pim^2–^ anions and three aqua ligands to complete a distorted MgO_6_ octahedra with the Mg–O distances in the range of 1.999 (3)–2.156 (2) Å. The *trans*– and *cisoid*– O–Mg–O angles lie in the region 81.4 (1)–98.7 (1)° and 168.5 (1)– 171.8 (1)°.The Mg coordination sphere in (I) is similar to that observed in Mg_2_(H_2_O)_6_(BTEC) (Liu *et al.*, 2009). The Mg^2+^ ions are bridged by the pimelate anions, forming the polymeric layers parallel to (100) (Fig. 2). When the Mg atom and the pim^2–^ anions are treated as 3– nodes, the two-dimensional layers can be best described as (4.8^2^) topological network. Intermolecular O—H···O hydrogen bonds (Table 1) between the aqua ligand and carboxylate oxygen atoms make a contribution to stabilization of the three-dimensional framework.

### Experimental

Dropwise addition of 1 *M* NaOH (1.0 ml) to a stirred aqueous solution of (0.1248 g, 0.5 mmol) MgSO_4_.7H_2_O in 5.0 ml H_2_O produced pale-white Mg(OH)_2_.xH_2_O precipitate, which was separated by centrifugation and washed with distilled water several times until no detectable SO_4_^2–^ anions in the supernatant. Subsequently, the 0.0815 g (0.5 mmol) pimelic acid was dissolved completely with 15 ml H_2_O, and then the precipitate was added. The resulting mixture was further stirred for 30 min and then filtered. The white filtrate (pH = 5.70) was allowed to stand at room temperature. Slow evaporation for several days afforded colourless platelet-like crystals.

### Refinement

H atoms bounded to C atoms were palced in geometrically calculated position and were refined using a riding model, with *U*_iso_(H) = 1.2 *U*_eq_(C). H atoms attached to O atoms were found in a difference Fourier synthesis and were refined using a riding model, with the O—H distances fixed as initially found and with *U*_iso_(H) values set at 1.2 *U*eq(O).

### Crystal data

**Table:**

[Mg_2_(C_7_H_10_O_4_)_2_(H_2_O)_6_]	*F*(000) = 504
*M**_r_* = 236.51	*D*_x_ = 1.474 Mg m^−^^3^
Monoclinic, *P*2_1_/*c*	Mo *K*α radiation, λ = 0.71073 Å
Hall symbol: -P 2ybc	Cell parameters from 6164 reflections
*a* = 14.311 (3) Å	θ = 3.4–27.4°
*b* = 8.2080 (16) Å	µ = 0.18 mm^−^^1^
*c* = 9.1280 (18) Å	*T* = 293 K
β = 96.22 (3)°	Platelet, colourless
*V* = 1065.9 (4) Å^3^	0.1 × 0.1 × 0.1 mm
*Z* = 4	

### Data collection

**Table:**

Rigaku R-AXIS RAPID diffractometer	1880 independent reflections
Radiation source: fine-focus sealed tube	1440 reflections with *I* > 2σ(*I*)
graphite	*R*_int_ = 0.040
Detector resolution: 0 pixels mm^-1^	θ_max_ = 25.0°, θ_min_ = 3.4°
ω scans	*h* = −16→17
Absorption correction: multi-scan (*ABSCOR*; Higashi, 1995)	*k* = −9→9
*T*_min_ = 0.982, *T*_max_ = 0.982	*l* = −10→10
8118 measured reflections	

### Refinement

**Table:**

Refinement on *F*^2^	Primary atom site location: structure-invariant direct methods
Least-squares matrix: full	Secondary atom site location: difference Fourier map
*R*[*F*^2^ > 2σ(*F*^2^)] = 0.046	Hydrogen site location: inferred from neighbouring sites
*wR*(*F*^2^) = 0.121	H-atom parameters constrained
*S* = 1.16	*w* = 1/[σ^2^(*F*_o_^2^) + (0.0228*P*)^2^ + 2.4599*P*] where *P* = (*F*_o_^2^ + 2*F*_c_^2^)/3
1880 reflections	(Δ/σ)_max_ < 0.001
136 parameters	Δρ_max_ = 0.34 e Å^−^^3^
0 restraints	Δρ_min_ = −0.45 e Å^−^^3^

### Special details

**Table:**

Geometry. All e.s.d.'s (except the e.s.d. in the dihedral angle between two l.s. planes) are estimated using the full covariance matrix. The cell e.s.d.'s are taken into account individually in the estimation of e.s.d.'s in distances, angles and torsion angles; correlations between e.s.d.'s in cell parameters are only used when they are defined by crystal symmetry. An approximate (isotropic) treatment of cell e.s.d.'s is used for estimating e.s.d.'s involving l.s. planes.
Refinement. Refinement of *F*^2^ against ALL reflections. The weighted *R*-factor *wR* and goodness of fit *S* are based on *F*^2^, conventional *R*-factors *R* are based on *F*, with *F* set to zero for negative *F*^2^. The threshold expression of *F*^2^ > σ(*F*^2^) is used only for calculating *R*-factors(gt) *etc*. and is not relevant to the choice of reflections for refinement. *R*-factors based on *F*^2^ are statistically about twice as large as those based on *F*, and *R*- factors based on ALL data will be even larger.

### Fractional atomic coordinates and isotropic or equivalent isotropic displacement parameters (Å^2^)

**Table:**

	*x*	*y*	*z*	*U*_iso_*/*U*_eq_	
Mg	0.83863 (7)	0.14609 (13)	0.01196 (11)	0.0200 (3)	
O1	0.84242 (17)	−0.0886 (3)	−0.0455 (3)	0.0360 (6)	
O2	0.87832 (17)	−0.3395 (3)	−0.0967 (3)	0.0329 (6)	
C1	0.8253 (2)	−0.2167 (4)	−0.1155 (4)	0.0236 (7)	
C2	0.7382 (2)	−0.2240 (5)	−0.2254 (4)	0.0300 (8)	
H2A	0.7349	−0.3299	−0.2729	0.036*	
H2B	0.7429	−0.1422	−0.3010	0.036*	
C3	0.6484 (2)	−0.1956 (5)	−0.1530 (4)	0.0328 (8)	
H3A	0.6418	−0.2815	−0.0820	0.039*	
H3B	0.6536	−0.0930	−0.1000	0.039*	
C4	0.5610 (2)	−0.1918 (5)	−0.2641 (4)	0.0331 (9)	
H4A	0.5639	−0.0966	−0.3265	0.040*	
H4B	0.5609	−0.2874	−0.3265	0.040*	
C5	0.4696 (2)	−0.1871 (5)	−0.1933 (4)	0.0325 (9)	
H5A	0.4668	−0.2815	−0.1300	0.039*	
H5B	0.4690	−0.0906	−0.1321	0.039*	
C6	0.3837 (2)	−0.1858 (5)	−0.3061 (4)	0.0315 (9)	
H6A	0.3897	−0.2728	−0.3764	0.038*	
H6B	0.3823	−0.0836	−0.3597	0.038*	
C7	0.2909 (2)	−0.2061 (4)	−0.2417 (3)	0.0228 (7)	
O3	0.28052 (15)	−0.1400 (3)	−0.1210 (2)	0.0258 (5)	
O4	0.22845 (16)	−0.2917 (3)	−0.3134 (2)	0.0334 (6)	
O5	0.92533 (15)	0.0681 (3)	0.2049 (2)	0.0235 (5)	
H5C	0.9043	−0.0086	0.2529	0.028*	
H5D	0.9462	0.1397	0.2650	0.028*	
O6	0.96828 (14)	0.1863 (3)	−0.0827 (2)	0.0248 (5)	
H6C	1.0161	0.2242	−0.0385	0.030*	
H6D	0.9957	0.1165	−0.1271	0.030*	
O7	0.86395 (18)	0.3864 (3)	0.0818 (3)	0.0362 (6)	
H7A	0.8443	0.3779	0.1634	0.043*	
H7B	0.8941	0.4666	0.0795	0.043*	

### Atomic displacement parameters (Å^2^)

**Table:**

	*U*^11^	*U*^22^	*U*^33^	*U*^12^	*U*^13^	*U*^23^
Mg	0.0191 (5)	0.0224 (6)	0.0185 (5)	0.0025 (4)	0.0021 (4)	−0.0003 (4)
O1	0.0318 (14)	0.0282 (14)	0.0459 (15)	0.0026 (11)	−0.0047 (12)	−0.0147 (12)
O2	0.0375 (14)	0.0289 (14)	0.0314 (13)	0.0097 (12)	0.0004 (11)	−0.0011 (11)
C1	0.0207 (16)	0.0225 (18)	0.0287 (17)	0.0000 (14)	0.0071 (14)	−0.0023 (15)
C2	0.0237 (17)	0.037 (2)	0.0285 (18)	−0.0024 (16)	−0.0003 (14)	−0.0087 (16)
C3	0.0236 (18)	0.042 (2)	0.0330 (19)	−0.0024 (16)	0.0022 (15)	−0.0051 (17)
C4	0.0214 (17)	0.045 (2)	0.0327 (19)	−0.0045 (16)	0.0038 (15)	−0.0068 (17)
C5	0.0204 (17)	0.044 (2)	0.0332 (19)	−0.0015 (16)	0.0028 (15)	−0.0064 (18)
C6	0.0221 (17)	0.049 (2)	0.0247 (18)	−0.0026 (16)	0.0076 (14)	−0.0054 (17)
C7	0.0187 (16)	0.0303 (19)	0.0191 (16)	−0.0001 (14)	0.0000 (13)	0.0031 (15)
O3	0.0221 (12)	0.0362 (14)	0.0192 (11)	−0.0031 (10)	0.0033 (9)	−0.0069 (10)
O4	0.0239 (12)	0.0552 (17)	0.0210 (12)	−0.0119 (12)	0.0022 (10)	−0.0101 (12)
O5	0.0267 (12)	0.0217 (12)	0.0217 (11)	−0.0028 (10)	0.0004 (9)	0.0009 (10)
O6	0.0176 (11)	0.0317 (13)	0.0251 (12)	0.0046 (10)	0.0017 (9)	0.0006 (10)
O7	0.0553 (17)	0.0227 (13)	0.0338 (14)	−0.0023 (12)	0.0204 (12)	0.0000 (11)

### Geometric parameters (Å, °)

**Table:**

Mg—O1	1.999 (3)	C4—H4B	0.9700
Mg—O4^i^	2.023 (2)	C5—C6	1.517 (5)
Mg—O3^ii^	2.066 (2)	C5—H5A	0.9700
Mg—O7	2.093 (3)	C5—H5B	0.9700
Mg—O5	2.140 (2)	C6—C7	1.518 (4)
Mg—O6	2.156 (2)	C6—H6A	0.9700
Mg—H7A	2.3478	C6—H6B	0.9700
O1—C1	1.241 (4)	C7—O3	1.251 (4)
O2—C1	1.263 (4)	C7—O4	1.263 (4)
C1—C2	1.514 (5)	O3—Mg^ii^	2.066 (2)
C2—C3	1.525 (5)	O4—Mg^iii^	2.023 (2)
C2—H2A	0.9700	O5—H5C	0.8407
C2—H2B	0.9700	O5—H5D	0.8365
C3—C4	1.523 (5)	O6—H6C	0.8177
C3—H3A	0.9700	O6—H6D	0.8245
C3—H3B	0.9700	O7—H7A	0.8268
C4—C5	1.521 (4)	O7—H7B	0.7876
C4—H4A	0.9700		
			
O1—Mg—O4^i^	91.82 (12)	C2—C3—H3B	109.1
O1—Mg—O3^ii^	98.66 (11)	H3A—C3—H3B	107.8
O4^i^—Mg—O3^ii^	95.83 (10)	C5—C4—C3	113.6 (3)
O1—Mg—O7	168.45 (11)	C5—C4—H4A	108.8
O4^i^—Mg—O7	94.85 (11)	C3—C4—H4A	108.8
O3^ii^—Mg—O7	90.04 (10)	C5—C4—H4B	108.8
O1—Mg—O5	84.14 (10)	C3—C4—H4B	108.8
O4^i^—Mg—O5	171.83 (10)	H4A—C4—H4B	107.7
O3^ii^—Mg—O5	91.81 (9)	C6—C5—C4	112.5 (3)
O7—Mg—O5	88.03 (10)	C6—C5—H5A	109.1
O1—Mg—O6	89.61 (10)	C4—C5—H5A	109.1
O4^i^—Mg—O6	86.98 (10)	C6—C5—H5B	109.1
O3^ii^—Mg—O6	171.16 (11)	C4—C5—H5B	109.1
O7—Mg—O6	81.36 (10)	H5A—C5—H5B	107.8
O5—Mg—O6	85.89 (9)	C5—C6—C7	114.5 (3)
O1—Mg—H7A	159.3	C5—C6—H6A	108.6
O4^i^—Mg—H7A	107.8	C7—C6—H6A	108.6
O3^ii^—Mg—H7A	73.4	C5—C6—H6B	108.6
O7—Mg—H7A	20.4	C7—C6—H6B	108.6
O5—Mg—H7A	77.2	H6A—C6—H6B	107.6
O6—Mg—H7A	97.8	O3—C7—O4	123.5 (3)
C1—O1—Mg	160.5 (2)	O3—C7—C6	119.1 (3)
O1—C1—O2	121.7 (3)	O4—C7—C6	117.4 (3)
O1—C1—C2	118.5 (3)	C7—O3—Mg^ii^	126.7 (2)
O2—C1—C2	119.9 (3)	C7—O4—Mg^iii^	147.7 (2)
C1—C2—C3	112.2 (3)	Mg—O5—H5C	116.3
C1—C2—H2A	109.2	Mg—O5—H5D	117.6
C3—C2—H2A	109.2	H5C—O5—H5D	107.9
C1—C2—H2B	109.2	Mg—O6—H6C	124.8
C3—C2—H2B	109.2	Mg—O6—H6D	124.5
H2A—C2—H2B	107.9	H6C—O6—H6D	95.2
C4—C3—C2	112.6 (3)	Mg—O7—H7A	97.5
C4—C3—H3A	109.1	Mg—O7—H7B	148.9
C2—C3—H3A	109.1	H7A—O7—H7B	109.5
C4—C3—H3B	109.1		

Symmetry codes: (i) −*x*+1, *y*+1/2, −*z*−1/2; (ii) −*x*+1, −*y*, −*z*; (iii) −*x*+1, *y*−1/2, −*z*−1/2.

### Hydrogen-bond geometry (Å, °)

**Table:**

*D*—H···*A*	*D*—H	H···*A*	*D*···*A*	*D*—H···*A*
O5—H5C···O2^iv^	0.84	1.92	2.741 (3)	165
O5—H5D···O6^v^	0.84	1.99	2.818 (3)	168
O6—H6C···O2^vi^	0.82	2.07	2.882 (3)	170
O6—H6D···O5^vi^	0.82	2.06	2.879 (3)	171
O7—H7A···O4^ii^	0.83	1.94	2.725 (3)	158
O7—H7B···O2^vii^	0.79	2.26	2.798 (3)	127

Symmetry codes: (iv) *x*, −*y*−1/2, *z*+1/2; (v) *x*, −*y*+1/2, *z*+1/2; (vi) −*x*+2, −*y*, −*z*; (ii) −*x*+1, −*y*, −*z*; (vii) *x*, *y*+1, *z*.
